# Multi-omics datasets of paddy field soils to support functional and ecological analysis of microbial communities

**DOI:** 10.1016/j.dib.2026.113298

**Published:** 2026-09-28

**Authors:** Qinghong Qian, Fangyu Mo, Fei Xu, Longjiang Fan

**Affiliations:** a Institute of Crop Science & Institute of Bioinformatics, Zhejiang University, Hangzhou, 310058, China; b Hainan Institute, Zhejiang University, Sanya, 572025, China

**Keywords:** Agroecosystem, Soil microbiome, Fertilization management, Metagenomics, Metatranscriptomics

## Abstract

Soil microbial communities are essential for ecosystem functioning and crop productivity. This dataset consists of 8 soil samples collected from four paddy field plots in Zhejiang, China, including organic and conventional management types during the winter fallow period under sunny conditions. For each plot, soil was sampled from two points at 10 cm depth, resulting in a total of 8 samples. Each sample was subjected to both metagenomic and metatranscriptomic sequencing, generating 16 datasets in total. DNA and RNA were extracted using standardized protocols, followed by Illumina PE150 sequencing. The metagenomic and metatranscriptomic data passed quality control and meet standard sequencing metrics, providing high-quality raw reads, assembled contigs, cDNA libraries, and gene expression matrices. These datasets are a reusable resource for studies on rice soil microbial communities and can support integration with other multi-omics data, such as proteomics or single-cell genomics, for broader investigations into soil microbial diversity and function.

Specifications Table

**Table:** 

Subject	Biology
Specific subject area	Soil metagenomics and metatranscriptomics of rice fields under organic and conventional management
Type of data	Table, Image, Raw read data (FASTQ)
Data collection	Soil samples were collected from experimental rice field plots used for agricultural breeding research during the winter fallow period under sunny conditions. Four plots were selected, including two organically managed plots and two conventionally fertilized plots. For each plot, soil was collected from two sampling points at a depth of 10 cm, resulting in 8 soil samples. Each sample was used for paired metagenomic and metatranscriptomic sequencing. DNA and RNA were extracted using standardized protocols, libraries were prepared, and paired-end sequencing was performed on an Illumina platform. Sequencing data passed standard quality control procedures.
Data source location	Institution: Ruian Agricultural breeding station City/Town/Region: Zhejiang Province Country: China
Data accessibility	Repository name: Genome Sequence Archive (GSA) Data identification number: CRA044660 Direct URL to data: https://ngdc.cncb.ac.cn/gsa/search?searchTerm=CRA044660
Related research article	None

## 1 Value of the Data

• High-quality paired metagenomic and metatranscriptomic datasets from rice field soils under organic and conventional fertilization provide a robust resource for exploring microbial diversity in agroecosystems.
• Comprehensive species annotation and sequencing quality metrics enable reuse for evaluating soil microbial community composition and assessing dataset reliability.
• Comparative information from soils under different management practices allows investigation of microbial community variation and ecological heterogeneity between and within treatments.
• The data support discovery of phage sequences, small open reading frames, and candidate antimicrobial peptides [1], with transcriptional data providing evidence for activity or expression of identified elements.
• Paired DNA and RNA datasets are suitable for integration with multi-omics studies, including proteomics, metabolomics, single-cell genomics [2], and transcriptomics [3], facilitating cross-study comparisons and meta-analyses in soil microbiology.

## 2 Background

Soil microbial communities are central components of agroecosystems and are closely associated with nutrient turnover, crop production, and soil ecological processes [4]. High-throughput sequencing has become a widely used approach for characterizing soil microbiomes, with metagenomics providing information on microbial genetic potential and metatranscriptomics capturing RNA-level activity from complex microbial communities. The combined use of these two approaches offers a framework for describing both the genomic content and transcriptional profiles of soil microbiomes under different field management conditions.

Paddy fields represent intensively managed agricultural systems, and fertilization practices can be associated with differences in soil microbial composition and activity. However, paired metagenomic and metatranscriptomic datasets from rice field soils under different fertilization regimes remain relatively limited in public data resources. This data article was compiled to provide paired DNA- and RNA-based sequencing data from organic and conventionally fertilized rice field soils during the winter fallow period. The dataset includes sequencing quality information and microbial species annotation profiles, providing reusable material for studies of rice soil microbiomes, cross-study comparison, multi-omics integration, phage or small-peptide mining, and computational workflow evaluation [4].

## 3 Data Description

This dataset comprises paired metagenomic and metatranscriptomic sequencing data from 8 rice field soil samples, including soils under organic and conventional fertilization management. Each soil sample was sequenced at both DNA and RNA levels, generating 16 datasets in total. The data provide high-quality reads suitable for downstream analyses of soil microbial communities.

Sequencing quality metrics include total bases, error rate, Q30 percentage, and GC content. Assembly statistics such as number of contigs, N50, and average contig length are also reported. These metrics are summarized in accompanying tables and figures, which provide an overview of sequencing depth, read quality, and assembly performance across all samples.

Microbial species annotation profiles derived from these datasets indicate the composition of dominant taxa in soils under different management practices, highlighting differences in community structure between organic and conventional soils. Tables and visual summaries allow users to examine the diversity and relative abundance patterns across samples.

Together, the dataset, quality metrics, and species profiles offer a comprehensive resource for the study of soil microbiomes. Figures and tables serve as supporting materials to familiarize readers with the dataset structure, data characteristics, and the range of microbial taxa captured Table 1.

**Table 1:** Sequencing quality and assembly statistics of host-filtered metagenomic and metatranscriptomic datasets.

Sample	Type	Total bases	Error%	Q30%	GC%	No. of contigs	N50 size (bp)	Average length (bp)
onventional_A1	MTS	1.68E+10	0.013	95.16	58.28	144,796	665	701.43
conventional_A2	MTS	1.91E+10	0.012	95.89	58.77	164,907	660	696.34
conventional_B1	MTS	1.19E+10	0.013	93.79	59.36	78,124	655	689.79
conventional_B2	MTS	1.99E+10	0.013	95.02	60.25	174,424	672	709.06
organic_A1	MTS	7.91E+09	0.012	95.68	58.42	96,940	691	733.90
organic_A2	MTS	1.79E+10	0.012	95.61	58.37	222,780	721	761.29
organic_B1	MTS	1.96E+10	0.012	95.50	59.36	250,004	691	728.86
organic_B2	MTS	1.69E+10	0.012	96.25	58.81	205,223	696	736.71
conventional_A1	MGS	9.84E+09	0.022	93.10	59.53	109,279	614	646.03
conventional_A2	MGS	7.19E+09	0.022	93.45	59.55	61,219	600	630.30
conventional_B1	MGS	6.46E+09	0.022	93.65	59.92	71,751	618	650.83
conventional_B2	MGS	8.55E+09	0.021	93.79	59.74	149,496	654	690.89
organic_A1	MGS	6.47E+09	0.022	93.62	58.84	132,125	697	739.66
organic_A2	MGS	8.39E+09	0.022	93.34	59.4	164,765	693	732.06
organic_B1	MGS	1.03E+10	0.022	93.71	59.61	176,312	658	703.52
organic_B2	MGS	1.02E+10	0.022	93.64	59.52	153,441	645	684.51

The table summarizes total bases, sequencing error rate, Q30 percentage, GC content, number of contigs, N50, and average contig length for paired metagenomic and metatranscriptomic datasets generated from 8 rice field soil samples. MTS and MGS refer to metagenomic and metatranscriptomic sequencing, respectively.

## 4 Experimental Design, Materials and Methods

### 4.1 Soil sampling and sequencing data generation

Soil samples were collected from experimental rice field plots used for agricultural breeding research. The sampling was conducted during the winter fallow period under sunny weather conditions. Four rice field plots were included, consisting of two organically managed plots and two conventionally fertilized plots. For each plot, two sampling points were selected, and soil was collected from a depth of 10 cm. This design generated 8 soil samples in total, including four organic soil samples and four conventional soil samples (Fig. 1).

**Fig. 1:**
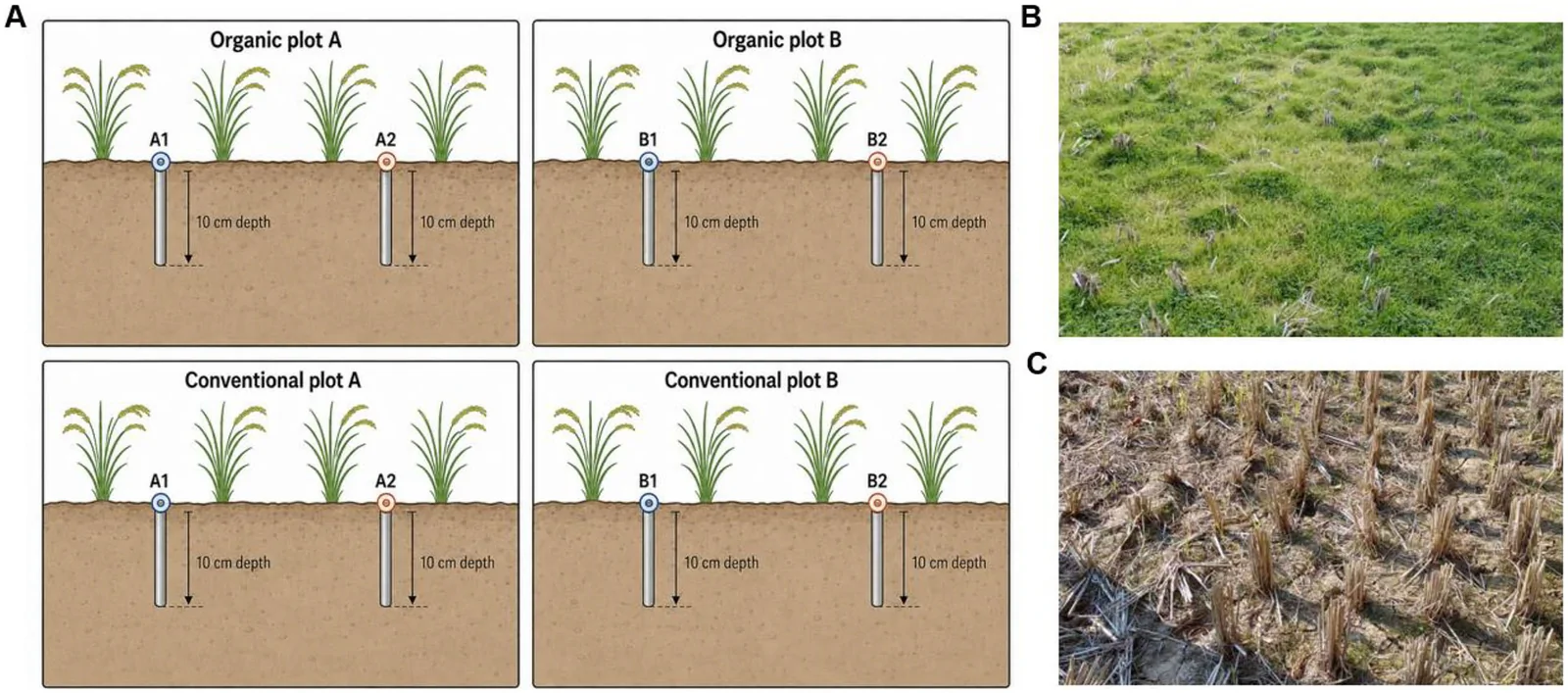
Overview of soil sampling design and rice field conditions. (A) Sampling scheme for 8 soil samples collected from four rice field plots, including two organically managed plots and two conventionally fertilized plots. Two sampling points were selected from each plot, and soil was collected at a depth of 10 cm. (B) Representative image of an organically managed rice field during the winter fallow period. (C) Representative image of a conventionally fertilized rice field during the winter fallow period.

Each soil sample was used for paired metagenomic and metatranscriptomic sequencing. Total DNA and total RNA were extracted from the same set of soil samples following standard soil nucleic acid extraction procedures. DNA samples were used for metagenomic library construction, and RNA samples were used for metatranscriptomic library construction after conversion into cDNA libraries. Libraries were sequenced using paired-end sequencing on an Illumina platform, generating 8 metagenomic datasets and 8 corresponding metatranscriptomic datasets.

After sequencing, raw reads were subjected to standard quality control procedures. Low-quality reads, adapter-contaminated reads, and reads containing ambiguous bases were removed. Host-related sequences were also filtered before the data quality and assembly statistics were summarized. The host-filtered sequencing datasets were evaluated using total bases, sequencing error rate, Q30 percentage, and GC content by FastQC 0.11.4. Assembly quality was described using the number of contigs, N50, and average contig length. These metrics were used to describe the sequencing quality and assembly characteristics of the paired metagenomic and metatranscriptomic data.

### 4.2 Species annotation and diversity-related data description

Microbial taxonomic annotation was performed using the processed metagenomic and metatranscriptomic datasets. Predicted gene sets were aligned against the NCBI non-redundant protein database (NR) using DIAMOND v0.9.19.120 [5], with an e-value threshold of 1e-5 and a minimum identity of 60%. Taxonomic information linked to NR entries was used to assign microbial taxa to annotated genes.

Species abundance was calculated by summing the abundances of genes assigned to the same species. Abundance profiles were then generated at Domain, Kingdom, Phylum, Class, Order, Family, Genus, and Species levels across all samples. The species-level annotation information is provided as tabular data and visualized as a heatmap [6,7] (Fig. 2).

**Fig. 2:**
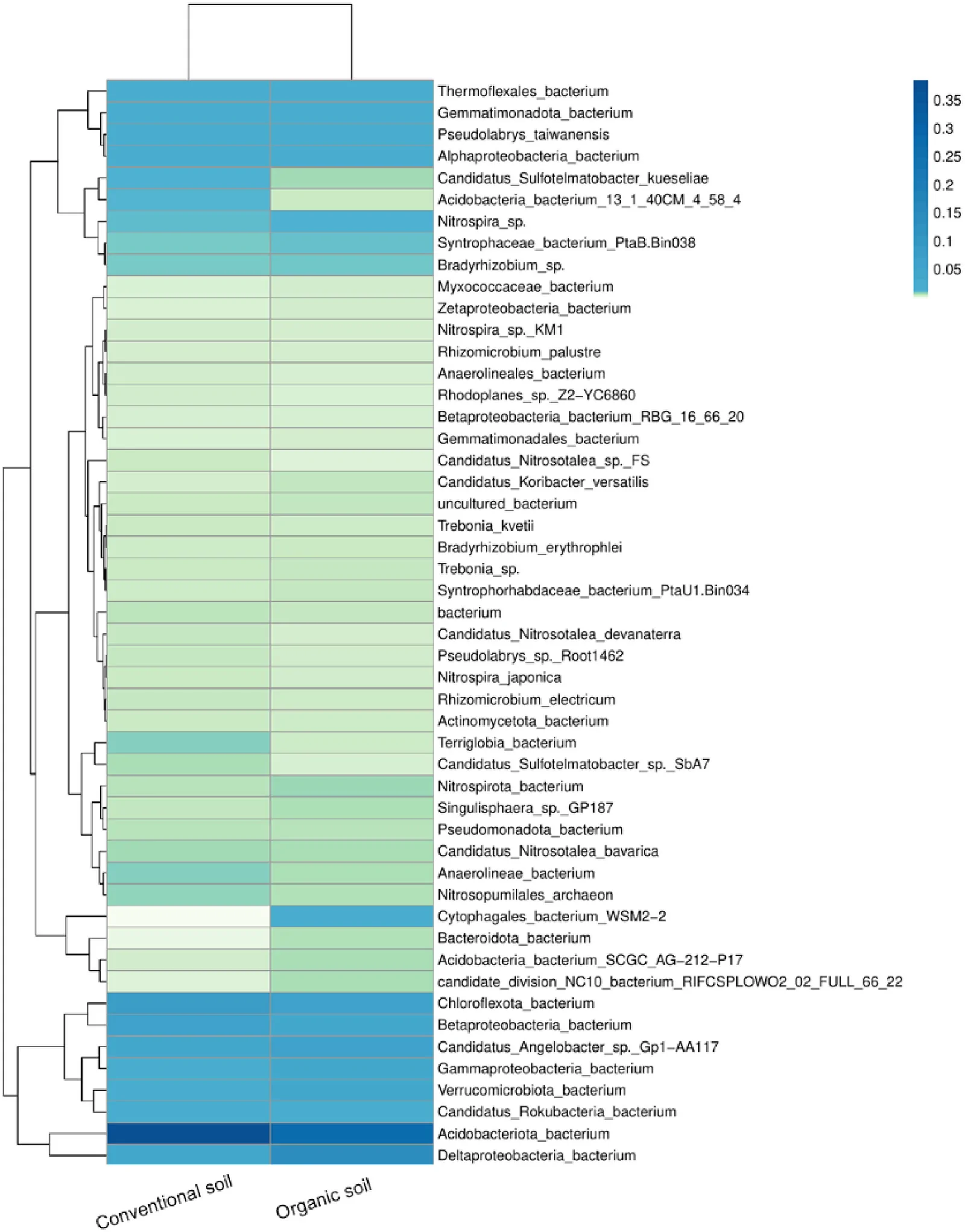
Microbial species annotation in rice field soils. Heatmap of species-level taxa across soil samples. Rows represent microbial taxa, columns represent soil management types (Organic soil, Conventional soil). Color intensity indicates relative abundance. Taxa are hierarchically clustered.

Diversity-related information was generated from the microbial annotation profiles to describe the species richness and diversity characteristics of the soil datasets. Chao1 and Shannon rarefaction curves were calculated for organic and conventional soil samples. The Chao1 rarefaction curve is shown in the left panel, and the Shannon rarefaction curve is shown in the right panel. In both panels, the x-axis represents sequences per sample, and the y-axis represents the corresponding alpha diversity measure. Error bars indicate variation among samples within each soil management type (Fig. 3).

**Fig. 3:**
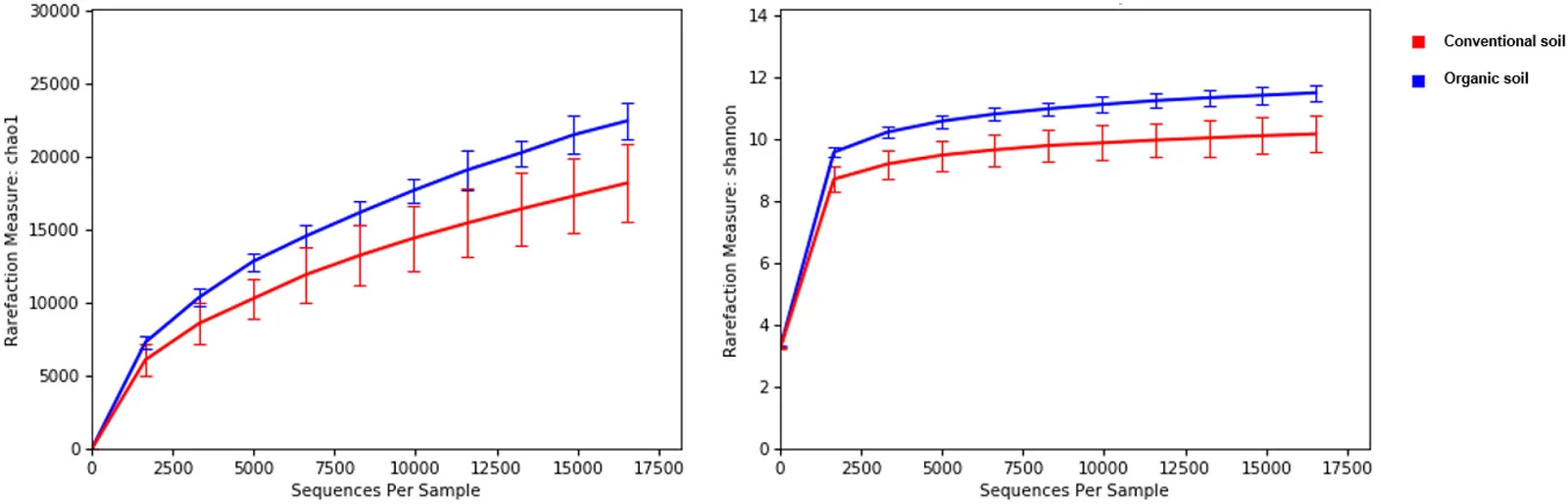
Alpha diversity rarefaction curves of rice field soil samples. Rarefaction curves of microbial alpha diversity in organic and conventional rice field soils. Chao1 richness index is shown on the left, and Shannon diversity index is shown on the right. The x-axis represents sequences per sample, and the y-axis represents the corresponding rarefaction measure. Error bars indicate variation among samples within each soil management type.

The species annotation table, heatmap, and rarefaction curves were included as auxiliary materials to describe the dataset and help readers understand the microbial taxa and diversity-related characteristics represented in the sequencing data.

## Limitations

The dataset comprises paired metagenomic and metatranscriptomic sequencing from 8 soil samples collected from four rice field plots. While the data are high quality, the limited number of samples may constrain the representation of broader spatial or temporal variability in rice field soils under different management practices.

## Ethics Statement

The authors have read and followed the ethical requirements for publication in Data in Brief. The current work does not involve human subjects, animal experiments, or data collected from social media platforms. The dataset was generated from rice field soil samples collected from experimental agricultural plots used for breeding research.

## CRediT Author Statement

**Qinghong Qian:** Sample collected, Formal analysis, Visualization, Writing - Original Draft. **Fangyu Mo:** Sample collected, Formal analysis, Visualization. **Fei Xu:** Sample collected. **Longjiang Fan:** Supervision, Writing - Review & Editing.

## Data Availability

NGDC), ChinaRice field soil metagenomic and metatranscriptomic datasets (Original data)
